# Computer‐Assisted Hip Arthroscopy for Portal Establishment and Suture Anchor Placement

**DOI:** 10.1002/atn2.70317

**Published:** 2026-10-19

**Authors:** Lingxing Jiang, Ming Hao, Mingxin Wang, Xiaoqi Kang, Zhanghua Tong, Jixiang Dong, Long Wang, Chunbao Li

**Affiliations:** ^1^ Department of Orthopedics Chinese PLA General Hospital Beijing People's Republic of China; ^2^ Graduate School of Chinese PLA General Hospital Beijing People's Republic of China; ^3^ Department of Orthopedics 923 Hospital of the Joint Logistic Support Force of the Chinese PLA Guangxi People's Republic of China

## Abstract

Hip arthroscopy is the gold standard for treating femoroacetabular impingement syndrome and acetabular labral tears. Portal establishment and suture anchor placement are crucial steps for ensuring the success of the surgery. However, because of the complex anatomy, deep location, and narrow joint space of the hip joint, the learning curve for establishing the hip arthroscopy approach and placing suture anchors for labral repair is arduous, with a high risk of iatrogenic cartilage and labral damage. This article describes a solution that applies computer‐assisted navigation to these 2 key steps. Based on the patient's preoperative computed tomography data, this technique enables the reconstruction of an individualized 3‐dimensional skeletal model, on which the clockface target of the anterolateral portal at the acetabular rim and the placement sites of suture anchors for labral repair are precisely planned. Intraoperatively, high‐precision spatial registration between the preoperative virtual plan and the patient's actual anatomical structure is achieved via 2‐dimensional to 3‐dimensional image registration. With the assistance of the navigation system, the surgeon can accurately establish the arthroscopic portal in a single attempt without fluoroscopy and safely perform acetabular suture anchor placement for subsequent labral suture.

## Graphical abstract

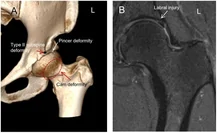

**VIDEO 1:** This video shows computer‐assisted hip arthroscopy for portal establishment and suture anchor placement, conducted by the Department of Orthopedics, Chinese PLA General Hospital. All authors declare no conflicts of interest associated with the authorship and publication of this work. Preoperative assessment included 3‐dimensional computed tomography reconstruction, which revealed a mixed‐type femoroacetabular impingement on the left hip, and magnetic resonance imaging, which showed a labral injury. Prior to surgery, surgical planning was performed on a dedicated planning system (Xinghang Orthopaedic Surgical Navigation System, Beijing, China), where the surgical portal target points and suture anchor placement sites were predetermined. The target positions for the anterolateral (AL) and midanterior (MA) portals were located within the hip joint space, adjacent to the 11‐o'clock position of the acetabulum, whereas the centers of the planned acetabular anchor placement sites were 3 to 4 mm from the acetabular rim, with an interanchor spacing of 5 to 6 mm. During navigation‐assisted intraoperative execution, the navigation interface displayed the extension line of the puncture instrument passing through the AL portal target; a long puncture guidewire was then inserted through the central channel of the guide bar, penetrating the joint capsule and entering the joint cavity, which was visualized on the navigation interface. Finally, a 30° arthroscope was inserted via the AL portal to obtain a clear surgical visual field. The MA portal was established using the same navigation‐assisted method: the navigation interface displayed the extension line of the puncture instrument passing through the MA portal target, a long puncture guidewire was inserted through the central channel of the guide bar to penetrate the joint capsule and enter the joint cavity, and then a 45° radiofrequency was placed into the MA portal to perform capsulotomy, which was visible from the AL portal. For navigation‐assisted acetabular drilling, a cannula was inserted via the distal anterolateral portal, with navigation guidance to accurately position the cannula at the preplanned anchor placement site; acetabular drilling and suture anchor placement were subsequently performed, followed by acetabular labral suture repair on completion of anchor implantation. Video content can be viewed at https://doi.org/10.1002/atn2.70317.

In the past 2 decades, hip arthroscopy has developed rapidly and achieved definite therapeutic effects.^1^, ^2^, ^3^ For femoroacetabular impingement (FAI) and acetabular labral tears, this technique is the gold standard.^4^, ^5^ However, portal establishment and suture anchor placement present arduous learning curves and high iatrogenic injury risks.^6^ Previous studies have shown that mastering hip arthroscopic portal establishment requires approximately 30 surgical procedures, achieving proficient hip arthroscopy requires 388 procedures, and accumulating 519 surgical experiences is necessary to achieve optimal surgical outcomes,^7^, ^8^ with a 12.3% overall reoperation rate.^8^

Iatrogenic chondral/labral injuries are common: 12.5% to 20% labral penetration during portal creation,^9^, ^10^ 73% chondral injury related to portal establishment,^11^ and 4.0% to 18.2% chondral injury from anchor placement,^12^ potentially leading to revision surgery.^6^, ^13^, ^14^ Computer‐assisted navigation can significantly improve the accuracy and safety of complex spatial manipulations^15^, ^16^, ^17^ and shorten the learning curve of sophisticated orthopaedic procedures.^18^ For hip arthroscopy in the treatment of FAI, which entails an extremely high technical difficulty, relevant research has mainly focused on cam osteochondroplasty.^19^, ^20^ Furthermore, compared with freehand operation, the navigation group achieves higher accuracy in cam osteochondroplasty and superior postoperative Nonarthritic Hip Score.^21^ However, its application in hip arthroscopic portal establishment and anchor placement has not been previously reported. This study describes a computer‐assisted technique to address these challenges (Table 1).

**TABLE 1:** Steps of Computer‐Assisted Hip Arthroscopy

1. Preoperative assessment	Identify bony deformities and labral injury areas based on preoperative CT and MRI.
2. Surgical planning	Plan the target regions for surgical portals and anchor placement sites in a 3D virtual model.
3. Navigation‐assisted intraoperative execution	
3.1. Tracker installation	Fix a reference frame on the ipsilateral iliac spine of the patient to ensure stable fixation.
3.2. Registration	Perform 2D to 3D image registration with fluoroscopy from at least 2 different angles.
3.3. Navigation‐assisted portal establishment	Mark the surgical instruments (half‐open cannula) and create the AL and MA portals according to the preoperatively planned target points.
3.4. Central compartment management	Perform debridement and labral repair in the central compartment and resect the pincer deformity and/or subspine deformity of the AIIS when indicated.
3.5. Navigation‐assisted labral repair	Guide acetabular drilling in real time under navigation to ensure the drill hole remains within the acetabular bone without penetration into the joint or pelvic cavity, followed by labral suture with suture anchors.

2D, 2‐dimensional; 3D, 3‐dimensional; AIIS, anterior inferior iliac spine; AL, anterolateral; CT, computed tomography; MA, midanterior; MRI, magnetic resonance imaging.

## SURGICAL TECHNIQUE

### Preoperative Imaging Evaluation

A detailed preoperative imaging evaluation is essential for computer navigation technology. Hip computed tomography and magnetic resonance imaging are usually required. Three‐dimensional (3D) computed tomography reconstruction can be used to diagnose FAI, assess the severity of these deformities, and determine the type of the anterior inferior iliac spine, thereby providing a basis for the resection of excessive bone during surgery (Figure 1). Magnetic resonance imaging helps evaluate the acetabular labrum, articular cartilage, and other soft‐tissue injuries.

**FIGURE 1:**
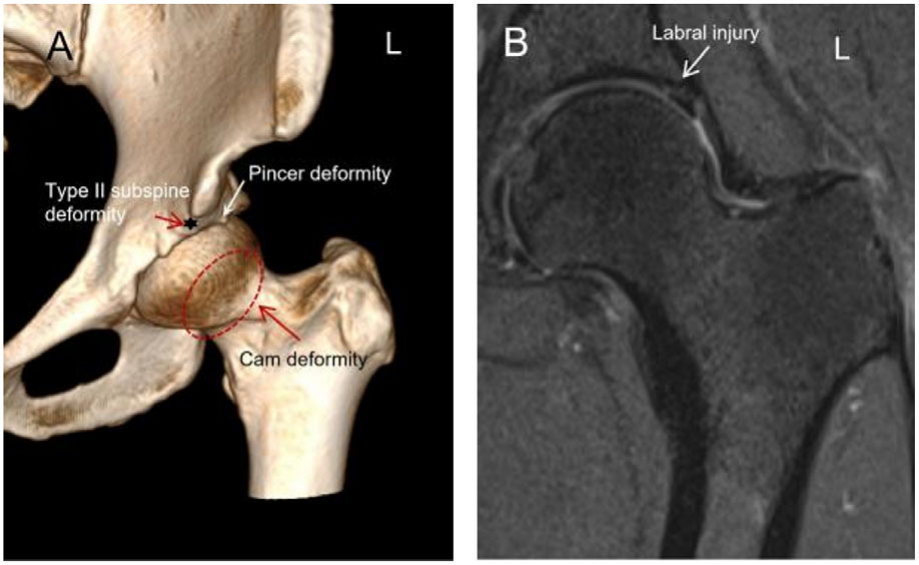
Preoperative assessment. (A) Preoperative 3D CT showing a cam deformity (red dashed circle), a pincer deformity (white arrow), and a type II subspine deformity of the AIIS (hexagram). (B) Oblique coronal MRI showing a labral injury (white arrow). (3D, 3‐dimensional; AIIS, anterior inferior iliac spine; CT, computed tomography; L, left hip; MRI, magnetic resonance imaging.)

### Preoperative Planning

Thin‐slice computed tomography (1 mm) Digital Imaging and Communications in Medicine data were imported into a preoperative planning system (Xinghang Orthopaedic Surgical Navigation System, Beijing Visual Medtech, Beijing, China). Using the acetabular clockface method (transverse acetabular ligament midpoint = 6‐o'clock),^22^ optimal anterolateral (AL) portal targets were set at the 11‐ to 12‐o'clock joint space, inferior to the acetabular rim on the oblique coronal plane. Because the establishment of the midanterior (MA) portal requires a combination of inside‐out and outside‐in techniques, the trajectory of the MA portal converges with that of the AL portal, and thus, the puncture target for the MA portal was determined to be consistent with that of the AL portal (Figure 2B). Anchor sites were planned 3 to 4 mm from the acetabular rim with an interanchor spacing of approximately 6 mm (Figure 2C).

**FIGURE 2:**
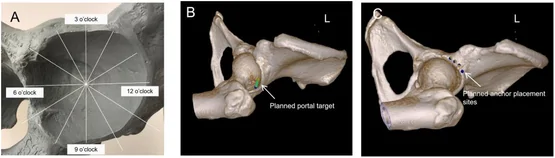
Preoperative planning. (A) Schematic of the acetabular clockface localization method, showing the 3‐, 6‐, 9‐, and 12‐o'clock positions of the L joint in the supine patient. (B) The targets for the arthroscopic AL and MA portals (green cylinders) are located at the joint space near the 11 to 12‐o'clock position of the acetabulum. (C) The centers of the planned acetabular anchor placement sites (yellow‐blue dots) are 3 to 4 mm from the acetabular rim with an interanchor spacing of 5 to 6 mm. (AL, anterolateral; L, left hip; MA, midanterior.)

### Intraoperative Registration

The patient was placed in the supine position on a traction table under general anesthesia. After routine skin preparation and sterile draping of the surgical field, an optical reference frame was percutaneously fixed to the iliac crest of the affected side (Figure 3A), and an infrared tracking camera (NDI Polaris Series, NDI, Canada) was positioned at an appropriate location at the patient's head end, ensuring that both the image registrator and the reference frame could be identified. Fluoroscopic images containing the image registrator were acquired using a C‐arm fluoroscope (Figure 3B), and the system achieved 2‐dimensional to 3‐dimensional registration (accuracy ≤1 mm).

**FIGURE 3:**
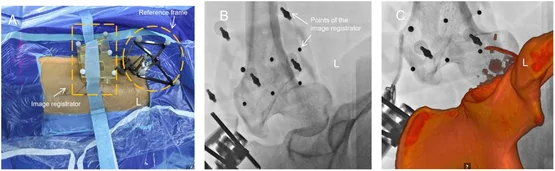
Intraoperative registration. (A) The reference frame (dashed circle) was fixed to the iliac crest of the affected side, and the image registrator (dashed square) was temporarily fixed to the surface of the left acetabulum. (B) C‐arm fluoroscopy was performed to ensure all 11 acquisition points of the image registrator were visible on the fluoroscopic image. (C) The 2D to 3D registration was performed on the navigation interface. (2D, 2‐dimensional; 3D, 3‐dimensional; L, left hip.)

### Navigation‐Assisted Portal Establishment

Using perineal postfree traction.^23^ First, a probe was used to localize the target at the body surface marking point of the AL portal. The puncture target was identified along the extension line of the probe, and the initial puncture angle and direction were determined (Figure 4A and B). A skin incision was made, and the extra‐articular soft tissues were bluntly dissected. A puncture guide rod calibrated by the navigation system was advanced toward the portal target, and the guide rod was gradually advanced until it contacted the hip joint capsule. Next, a long puncture guidewire was inserted through the central channel of the guide rod, penetrating the joint capsule and entering the joint cavity. Portal dilation was performed, and the puncture guide rod was further advanced to reach the target point (Figure 4C). It is important to note that during portal dilation, once a sudden loss of resistance is felt as the dilator passes through the joint capsule, advancement of the needle tip must be halted to prevent injury to the acetabular labrum and femoral head cartilage. A 30° arthroscope (Smith & Nephew) was then inserted to confirm a clear arthroscopic visual field and no injury to surrounding tissues (Video 1). The MA portal was established using the same puncture method under navigation guidance (Video 1).

**FIGURE 4:**
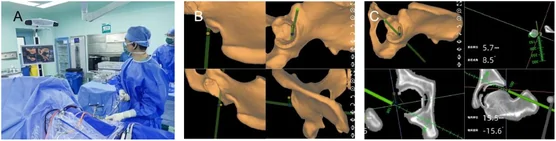
Navigation‐assisted portal establishment. (A) Navigation‐guided creation of the AL portal using a semiopen hollow guide bar (red arrow). (B) The navigation interface displaying the extension line of the puncture instrument (green transparent cylinder) passing through the AL portal target (blue dot). The cross arrow indicates the portal target. (C) The navigation interface showing the tip of the puncture instrument (green opaque cylinder) reaching the AL portal target (cross arrow) after portal establishment. (AL, anterolateral.)

### Navigation‐Assisted Labral Repair

A transverse anterior capsular incision between the AL and MA portals was made to explore the central compartment. A radiofrequency (Smith & Nephew, Andover, MA) ablator was used to debride soft tissues adjacent to the labrum, exposing the acetabular bone. Acetabuloplasty (Figure 5A and B) and anterior inferior iliac spine plasty (Figure 6A and B) were performed using a spherical burr (Smith & Nephew) to prepare a bony bed for labral repair. A distal anterolateral portal was used for anchor placement. First, calibration and registration of the anchor placement cannula were completed. Subsequently, the anchor placement cannula was positioned at the planned site via the distal anterolateral portal. The navigation system guided the surgeon to determine the safest anchor placement angle through its multiplanar reconstruction function and the scales on the instrument extension line (Figure 7A and B), and 2.3 mm anchors (Smith & Nephew) were placed after 20 mm depth‐limited drilling (Figure 8A and C, Video 1).

**FIGURE 5:**
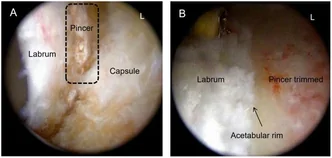
Management of pincer deformity. Arthroscopic view via the AL portal of the L joint in a supine patient. (A) The edge of the pincer deformity showing an irregular curved morphology. (B) The pincer deformity was fully resected, with a smooth acetabular rim achieved. (AL, anterolateral; L, left hip.)

**FIGURE 6:**
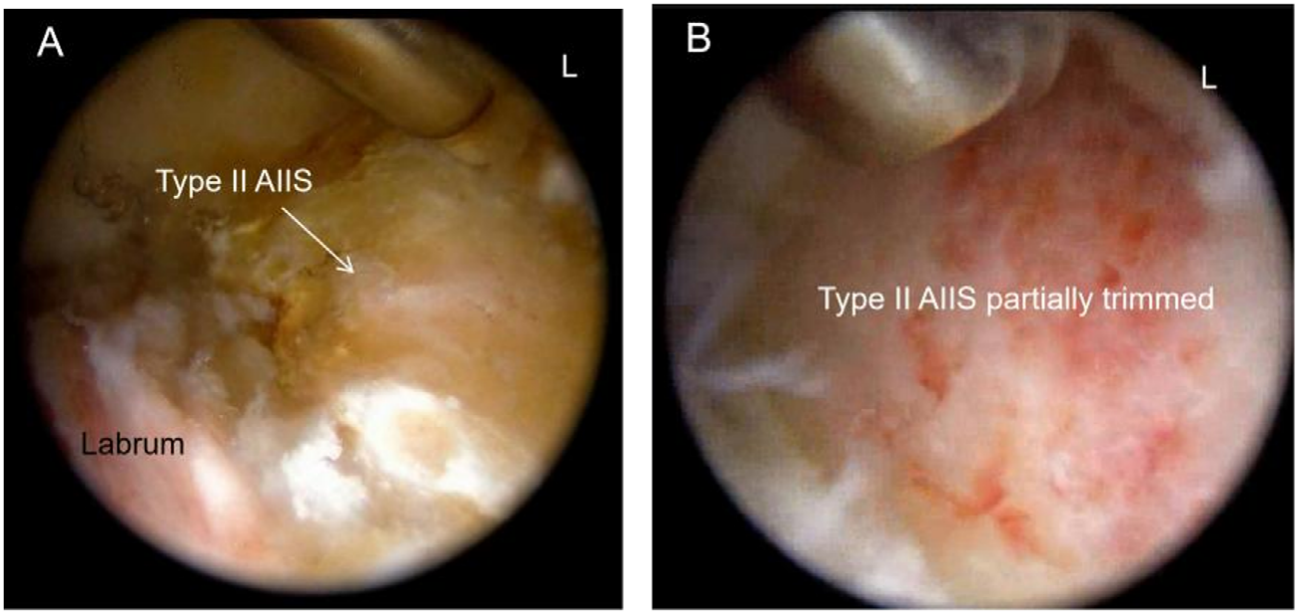
Management of the AIIS deformity. Arthroscopic view via the MA portal of the L joint in a supine patient (A) A typical type II AIIS subspine deformity. (B) The type II deformity was completely corrected. (AIIS, anterior inferior iliac spine; L, left hip; MA, midanterior.)

**FIGURE 7:**
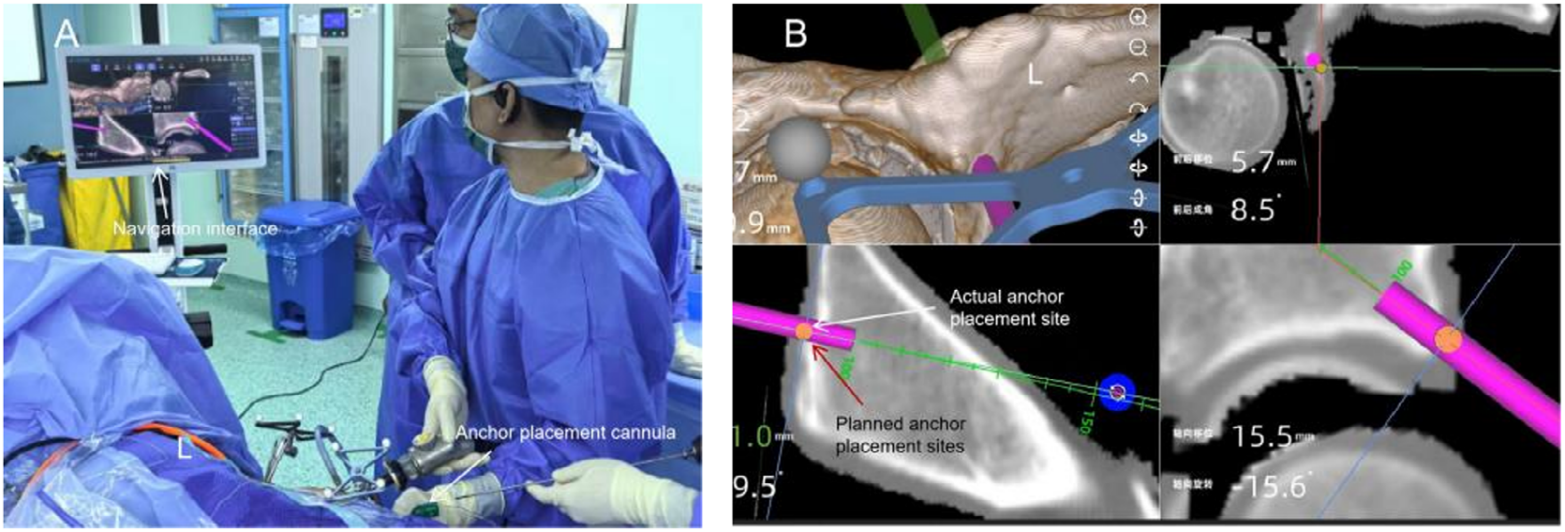
Navigation‐assisted acetabular drilling. (A) A cannula was inserted via the DALA portal, with navigation guidance for cannula positioning at the planned anchor placement site (orange dot). (B) The purple cylinder indicates the position of the anchor placement cannula; the orange dot denotes the planned anchor placement site, with the center of the cross (arrow) corresponding to the actual site; the cannula is precisely aligned with the planned anchor placement site. (DALA, distal anterolateral.)

**FIGURE 8:**
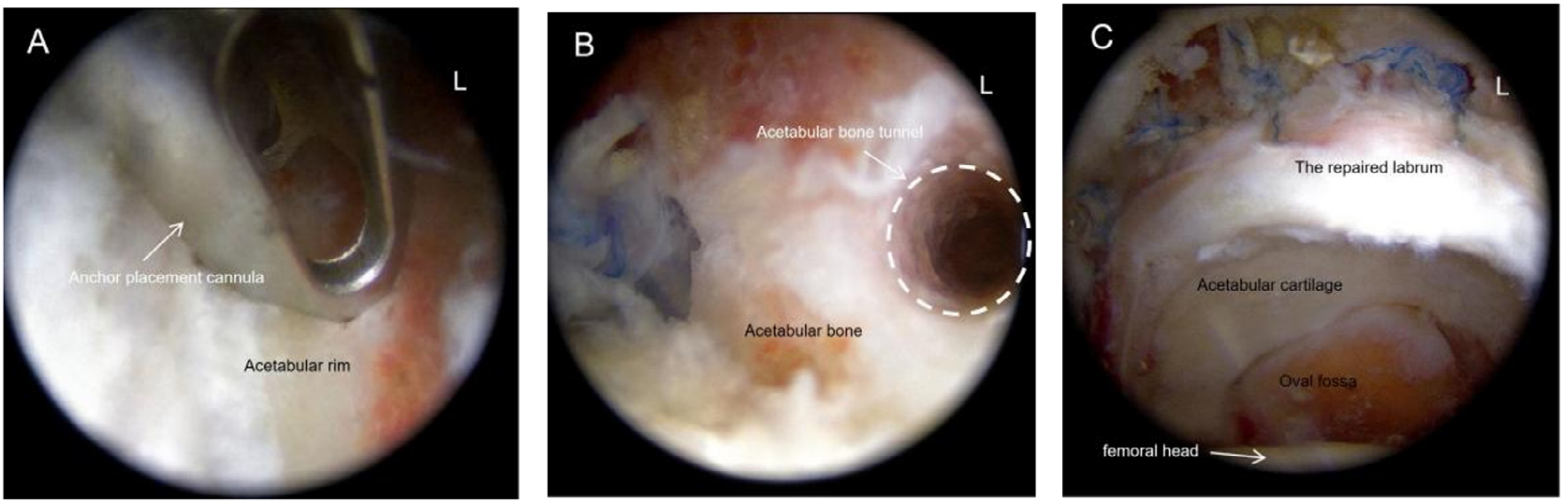
Management of labral injury. (A) Arthroscopic view via the AL portal of the L joint in a supine patient Navigation‐assisted localization of the acetabular anchor placement cannula at the acetabular rim. (B) The bone tunnel (white dashed circle) is positioned appropriately without cortical penetration. (C) The labrum presented good morphology and tension after suturing, with restored anatomical alignment to the acetabulum. (AL, anterolateral; L, left hip.)

After completion of the injured labrum repair, other pathological lesions in the central compartment were explored and managed. Traction on the affected extremity was then slowly released. The arthroscope was reinserted via the AL portal, and a longitudinal incision was made in the anterior joint capsule via the distal anterolateral portal until the cam lesion at the femoral head‐neck junction was fully exposed. The cam lesion was resected using a burr, and femoral head‐neck plasty was performed (Figure 9A and C). Arthroscopic observation confirmed complete resolution of FAI. Side‐to‐side suture of the incised joint capsule was then performed (Figure 10A and B). The surgical incision was closed, and the operation was completed.

**FIGURE 9:**
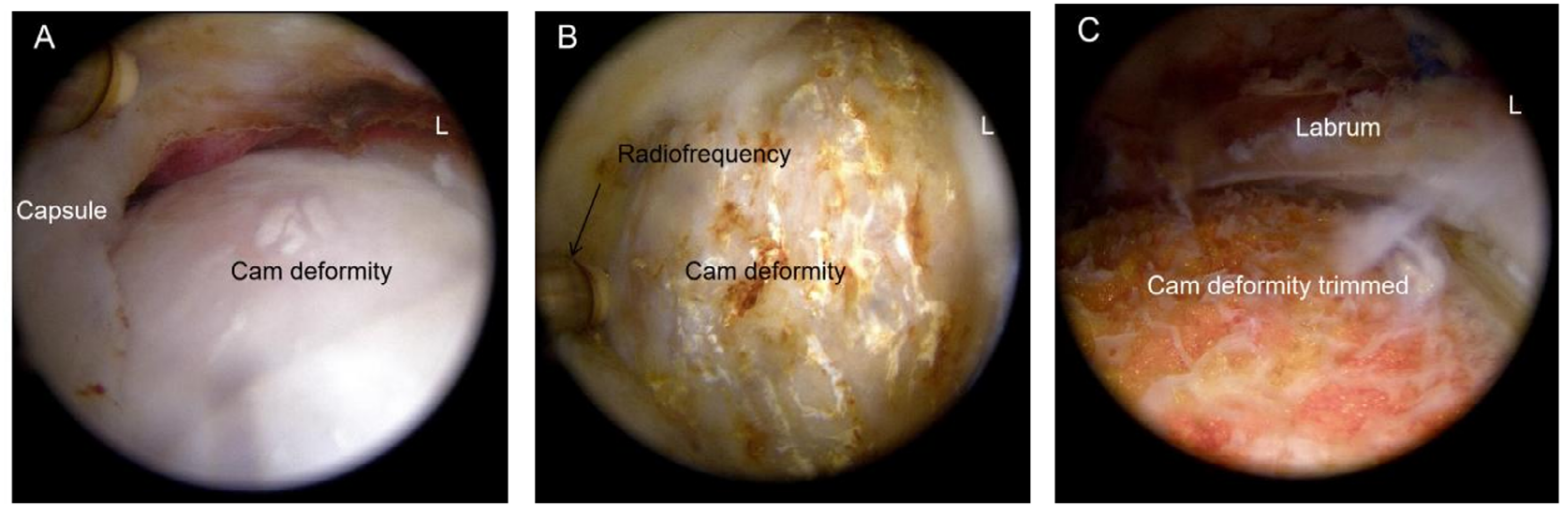
Management of cam deformity. Arthroscopic view via the AL portal of the L joint in a supine patient. (A) Exposure of the cam deformity. (B) The cam deformity was marked using radiofrequency ablation. (C) The cam deformity was completely resected without impingement during hip flexion. (AL, anterolateral; L, left hip.)

**FIGURE 10:**
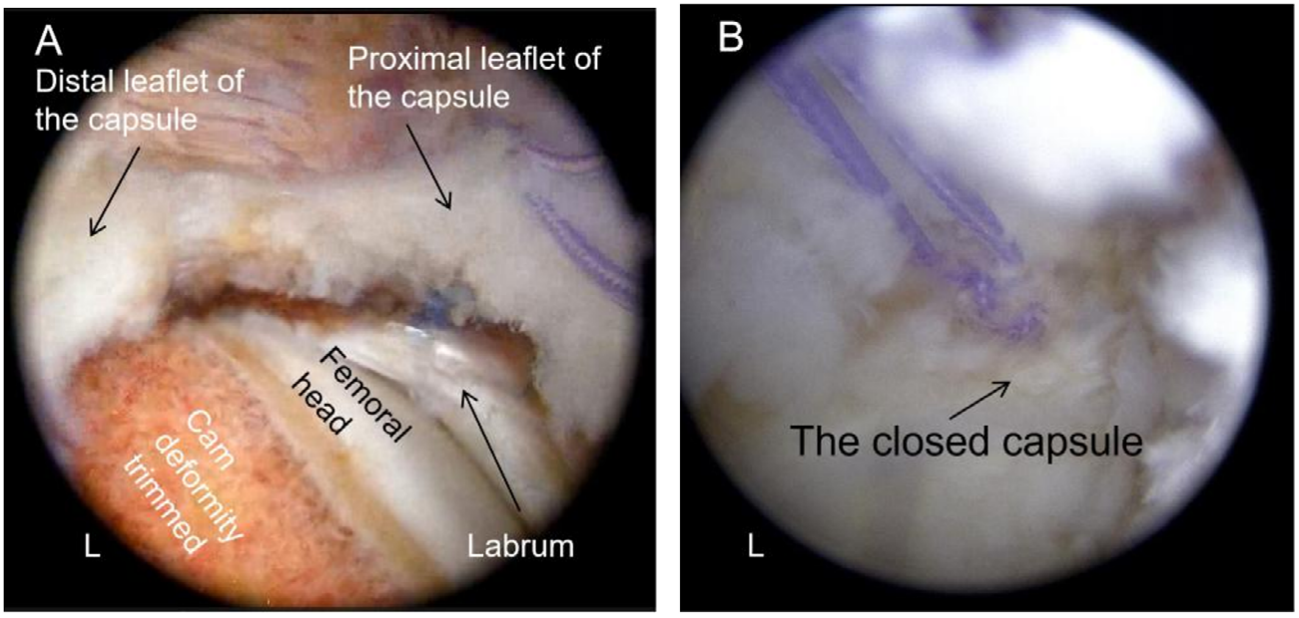
Management of the joint capsule. Arthroscopic view via the AL portal of the L joint in a supine patient. The joint capsule before closure. (A) The cam deformity at the head‐neck junction was sufficiently resected, and the repaired labrum restored the sealing effect. The joint capsule after closure. (B) Capsular closure was performed at the end of the operation to minimize impairment of joint stability. (AL, anterolateral; L, left hip.)

## DISCUSSION

Hip arthroscopy is technically demanding, with portal establishment and acetabular anchor placement posing arduous learning curves and high iatrogenic injury risks. This study is the first to validate computer navigation for these 2 critical steps, offering a solution to address conventional hip arthroscopy's limitations.

As the primary observational portal, the AL portal is challenging for novices, requiring at least 30 surgeries to master.^7^ Conventional fluoroscopy‐guided portal creation relies on 2‐dimensional imaging to infer 3‐dimensional instrument trajectories,^24^, ^25^ which is essentially a blind puncture maneuver and may lead to iatrogenic labral/cartilage injuries. Ultrasound‐guided techniques demand additional learning and have poor bony visualization in complex cases, whereas fluoroscopy‐free methods are reserved for experienced surgeons.^23^, ^26^, ^27^ In contrast, our navigation system provides real‐time 3D visualization of instruments, enabling precise alignment with preplanned targets and eliminating reliance on spatial imagination.

Anchor placement for labral repair is equally challenging because of the hip's deep, irregular anatomy. Freehand placement, even with fluoroscopy, struggles to control anchor depth and cartilage distance, resulting in a 4.0% to 18.2% iatrogenic cartilage injury rate that may lead to revision surgery.^12^ Although small‐diameter anchors and arthroscopic monitoring reduce risks,^12^ they fail to address poor visualization and spatial manipulation difficulties. Our navigation technology achieves seamless integration of preoperative planning and intraoperative execution, with multiplanar feedback ensuring instruments stay within safe boundaries—an advantage over static fluoroscopy or freehand techniques. It also avoids additional operator radiation exposure during osteochondroplasty.

Beyond precision and safety, a prominent potential value of this technology is the significant reduction in the learning curve. With hundreds of surgeries needed to achieve low complication rates,^8^ navigation standardizes and visualizes key steps, helping novices build spatial cognition and reduce errors.

Notably, limitations exist: high initial costs restrict access to large tertiary hospitals, additional skin incisions are required for reference frames, and registration adds procedural time. It is critical to emphasize is that navigation is an auxiliary tool—surgeon clinical judgment and arthroscopic visualization remain indispensable to avoid errors (Tables 2 and 3).

**TABLE 2:** Pearls and Pitfalls

**Pearls**	**Pitfalls**
Preoperative planning of anchor placement sites and anterolateral portal targets should be performed based on the assessed labral injury and pincer deformity.	Discrepancies may exist between preoperative planning and intraoperative execution.
The optical reference frame and image registrator must be firmly fixed during registration to prevent displacement.	Displacement of the reference frame or image registrator will result in reduced accuracy of the navigation system.
Unobstructed visual contact should be maintained between the optical tracking camera, reference frame, and surgical instruments.	Obstruction of the camera's field of view by surgical staff or equipment during the operation will cause navigation interruption.
Surgeons must be familiar with the navigation interface and understand the spatial correspondence between virtual instruments, preplanned sites, and actual anatomical structures.	Over‐reliance on navigation as absolute instructions, while neglecting the surgeon's clinical judgment and direct arthroscopic observation.
Instrument angles should be fine tuned according to navigation feedback; drilling and anchor placement should only be performed after final confirmation under fluoroscopy or direct arthroscopic visualization.	After osteophyte resection, the position of the instrument tip may be inconsistent between the 3D virtual model and multiplanar views, which may mislead surgical judgment.

3D, 3‐dimensional.

**TABLE 3:** Technical Advantages and Current Limitations

**Advantages**	**Limitations**
Visualization of puncture targets and acetabular anchor placement sites is achieved, reducing the risk of iatrogenic chondral and labral injuries.	Preoperative 3D modeling fails to incorporate soft tissues, including cartilage and the labrum, and cannot simulate the processes of traction and lower‐limb internal rotation.
Postregistration reliance on repeated intraoperative fluoroscopic confirmation is reduced, lowering radiation exposure for both surgeons and patients.	Intraoperative procedural accuracy is influenced by multiple factors, including registration accuracy, instrument calibration, and surgical technique of the operator.
Visualization of puncture targets and acetabular anchor placement sites facilitates a shortened learning curve.	Additional skin incisions are required.
The anterior edge of the pincer deformity can be preoperatively planned, facilitating its intraoperative localization.	Specialized navigation equipment and processing software are mandatory, entailing high initial investment costs and occupying extra operating room space.

3D, 3‐dimensional.

This study shows that computer‐assisted navigation enables safe, precise hip arthroscopic portal establishment, and anchor placement. By leveraging individualized preoperative planning and real‐time 3D guidance, it improves surgical safety, precision, and reproducibility while reducing learning curves, holding broad clinical translation prospects.

## DISCLOSURES

The authors (L.J., M.H., M.W., X.K., Z.T., J.D., L.W., C.L.) declare that they have no known competing financial interests or personal relationships that could have appeared to influence the work reported in this paper.

## FUNDING

The study was supported by Beijing Natural Science Foundation (L252061).
